# Supplementary material for: Seroprevalence and risk factors associated with exposure to Leishmania infantum in dogs, in an endemic Mediterranean region
Source: PLoS One. 2021 Jan 4;16(1):e0244923. doi: 10.1371/journal.pone.0244923 (PMC7781377; doi:10.1371/journal.pone.0244923)
Supplement: Attachment — Submitted filename: rebuttal letter_AC.docx [file pone.0244923.s002.docx]

Dear Editor,

We were pleased to revise the manuscript previously entitled “Risk factors for canine leishmaniasis in kennel and owned dogs in an endemic Mediterranean region”.

In this revision, we decided to change the title in: “Seroprevalence and risk factors associated with exposure to *Leishmania infantum* in kennel and owned dogs, in an endemic Mediterranean region”.

In the revised manuscript, we have carefully considered the editor’s and reviewers’ suggestions and we edited the manuscript and added some paragraphs accordingly.

Following are our answers to each point raised by the Academic Editor and the reviewers.

We thank the Editor and all reviewers for their comments to the manuscript. We feel they were very helpful in the improvement of the paper.

We hope that, after the performed changes, the article meets the target and the requirements of your Journal.

Sincerely

Pasquale Rombolà

| **#** | **Editor’s comments** | **Authors’ answers** |
| --- | --- | --- |
| 1 | 1. Please ensure that your manuscript meets PLOS ONE's style requirements, including those for file naming. The PLOS ONE style templates can be found at  <https://journals.plos.org/plosone/s/file?id=wjVg/PLOSOne_formatting_sample_main_body.pdf> and <https://journals.plos.org/plosone/s/file?id=ba62/PLOSOne_formatting_sample_title_authors_affiliations.pdf> | We ensure that the manuscript meets all the PLOS ONE's style requirements |
| 2 | 2. As part of your revisions, please address the following requests: (1) explain how you were able to access the sera samples collected by the veterinarians. As part of this explanation, please state whether you obtained any type of permission or authorization to obtain access to these samples. (2) Kindly provide details about consent that dog owners provided prior to blood collection by veterinarians. | Thank you for this important comment.  1) We explained how the samples were selected in the beginning of the “sampling and data” paragraph of M&M section as follows: “…in accordance with a Regional Law, all dogs entering in kennels are compulsory tested for the presence of antibodies against L. infantum by sending blood samples to laboratories accredited under ISO/IEC 17025 (DGR 473/2010).”  1, 2) “The owned dogs were enrolled in specific research projects or sampled by veterinary practitioners for annual CanL screening, diagnosis or follow-up. (…) “Blood samples were collected for the following reasons: kennel admittance, annual screening, clinical suspicion, differential diagnosis and follow up after pharmacological treatment”(…) “The owned dogs were enrolled during routinely veterinary clinical activity”.  The consent is in charge of veterinarians, so we cannot provide this statement. Veterinarians authorize us to deal with data that are necessary for the required service (laboratory diagnosis, notification of positive samples, research scope etc.) |
| 3 | 3. We note that Figure 1 in your submission contain map images which may be copyrighted. All PLOS content is published under the Creative Commons Attribution License (CC BY 4.0), which means that the manuscript, images, and Supporting Information files will be freely available online, and any third party is permitted to access, download, copy, distribute, and use these materials in any way, even commercially, with proper attribution. For these reasons, we cannot publish previously copyrighted maps or satellite images created using proprietary data, such as Google software (Google Maps, Street View, and Earth). For more information, see our copyright guidelines: <http://journals.plos.org/plosone/s/licenses-and-copyright>.  We require you to either (1) present written permission from the copyright holder to publish these figures specifically under the CC BY 4.0 license, or (2) remove the figures from your submission:  3.1.    You may seek permission from the original copyright holder of Figure 1 to publish the content specifically under the CC BY 4.0 license.  We recommend that you contact the original copyright holder with the Content Permission Form (<http://journals.plos.org/plosone/s/file?id=7c09/content-permission-form.pdf>) and the following text:  “I request permission for the open-access journal PLOS ONE to publish XXX under the Creative Commons Attribution License (CCAL) CC BY 4.0 (<http://creativecommons.org/licenses/by/4.0/>). Please be aware that this license allows unrestricted use and distribution, even commercially, by third parties. Please reply and provide explicit written permission to publish XXX under a CC BY license and complete the attached form.”   Please upload the completed Content Permission Form or other proof of granted permissions as an "Other" file with your submission.   In the figure caption of the copyrighted figure, please include the following text: “Reprinted from [ref] under a CC BY license, with permission from [name of publisher], original copyright [original copyright year].”   3.2.    If you are unable to obtain permission from the original copyright holder to publish these figures under the CC BY 4.0 license or if the copyright holder’s requirements are incompatible with the CC BY 4.0 license, please either i) remove the figure or ii) supply a replacement figure that complies with the CC BY 4.0 license. Please check copyright information on all replacement figures and update the figure caption with source information. If applicable, please specify in the figure caption text when a figure is similar but not identical to the original image and is therefore for illustrative purposes only.  The following resources for replacing copyrighted map figures may be helpful:  USGS National Map Viewer (public domain): <http://viewer.nationalmap.gov/viewer/>  The Gateway to Astronaut Photography of Earth (public domain): <http://eol.jsc.nasa.gov/sseop/clickmap/>  Maps at the CIA (public domain): <https://www.cia.gov/library/publications/the-world-factbook/index.html> and <https://www.cia.gov/library/publications/cia-maps-publications/index.html>  NASA Earth Observatory (public domain): <http://earthobservatory.nasa.gov/>  Landsat: <http://landsat.visibleearth.nasa.gov/>  USGS EROS (Earth Resources Observatory and Science (EROS) Center) (public domain): [http://eros.usgs.gov/#](http://eros.usgs.gov/)  Natural Earth (public domain): <http://www.naturalearthdata.com/> | The image in Figure 1 is not copyrighted. The map was created using the ArcGis software, as declared in M&M. We are the owner of this software.  The layers we used in the map are two: Digital Elevation Model and the Italian administrative boundaries. They are open source data from the following sources (present in references): http://www.sinanet.isprambiente.it/it/sia-ispra/download-mais/dem20/view https://www.istat.it/it/archivio/222527  The ISPRA layer is open source under the "Italian Open Data License" (IODL) that is a Creative Commons license (CC BY 4.0) which makes it possible to freely reproduce, distribute, transmit and adapt data also for commercial purposes, provided the source is cited.  The ISTAT layer is open source under the "Italian Open Data License" (IODL) that is a Creative Commons license (CC BY 3.0) which makes it possible to freely reproduce, distribute, transmit and adapt data also for commercial purposes, provided the source is cited.  We upload as “other file” the two document that state this. |
| 4 | Please include captions for your Supporting Information files at the end of your manuscript, and update any in-text citations to match accordingly. Please see our Supporting Information guidelines for more information: <http://journals.plos.org/plosone/s/supporting-information> | We will update the following support information:  1 excel dataset used for the study. It was compressed as .rar. Name: S1 DTASET LEISH.rar |

Reviewer #1

|  | It is a generally well written manuscript, describing a seroprevalence study with risk factors assessment in a dog population of an endemic area for L. infantum zoonotic leishmaniasis in Italy. Although no striking novelty is brought, the study results’ contribution is valuable in epidemiological terms, mainly considering the number of dogs assessed and interesting results concerning the dynamics of the disease in the region. The work requires some writing edition and English revision. |  |
| --- | --- | --- |
| L 17-21 | **Abstract**: Requires a better restructuration for more objectivity. Please clarify your objectives, describe your results accordingly and consistently provide your conclusions. | We modified the abstract. Regarding the objectives, we modified it as follows:  “The aim of this study was to assess the influence of different individual, environmental and spatial risk factors on the dog exposure to *L. infantum* and to estimate the seroprevalence among owned and kennel dogs, in the Lazio region (central Italy), where canine leishmaniasis is endemic” |
| L 24 | Please describe the acronym IFAT the first time it appears. | Thanks, we modified it. |
| L 32-34 | For instance, the statement “This study confirmed the importance of the kennels as useful component for CanL active and passive surveillance.” is a bit vague and not exactly in accordance with the objectives of the study. If evaluating kennels was among the study’s objectives, it need to be clear. | Reviewer is right. We reduced the impact of kennels in many parts of the paper. It was not an objective of the paper, as it was not designed for this. It was a comment raised during the discussion of the results.  We modified the order of the sentences and the sentence as follows:  “The role of kennels as a key component for CanL active and passive surveillance was also highlighted. This study confirmed the endemicity of CanL in the Lazio region and focused some factors that can influence the seropositivity of dogs in a Mediterranean region.” |
|  | **Introduction**: Please organize your ideas by separating statements and information on humans and animals’ leishmaniasis clearly, preferably in separate paragraphs. Please use indentation for paragraphs and avoid an excessive use of parentheses. | We modified the structure of the section according to your observations. |
| L 47 | Please replace “restricted” by a more appropriate word for the idea; perhaps “concentrated”. | Thanks, we modified it. |
| L. 51 | Please observe your statement: “According to some estimates, in southern Europe there are 700 new human cases of ZVL per year, about 200 of those cases occurring annually in Italy”. You are using references from 2010 and 2011, which is not consistent with the idea of “new cases”. Please observe the use of proper references and amend accordingly here and in the whole manuscript. | We modified in this way, changing the references:  “The estimated annual incidence of visceral leishmaniasis (VL) in the WHO European Region varies between 1100 and 1900 cases [6]. Italy is among the most affected countries, together with Georgia, Spain, Albania, Turkey, Tajikistan and Azerbaijan, showing an estimated annual VL incidence from 160 to 240. In Italy, the most relevant endemic foci are in southern-central regions such as Tuscany, Sicily, Campania and Sardinia [6]. Notwithstanding, Italy provided the first evidence in Europe of the emergence and northward spread of VL as probable effect of climate change [7]” |
| L. 52-53 | Please observe the phrase: “CanL causes a spectrum of clinical signs that varies greatly from asymptomatic/mild to a very severe disease.” CanL is the disease, it does not cause it. The clinical signs of an infected dog can vary from absent – in subclinical infections – to numerous and intense. The disease can vary from unapparent to mild or very severe. Amend accordingly. | Thanks, we modified it. |
| L. 55 | Please replace “are” by a more appropriate “can be”. | Thanks, we modified it. |
| L. 58-60 | There are many differences between IFAT and ELISA for this purpose, depending on the antigen and many other aspects, and these varies considerably. So, the statement is a bit too generalized. Please amend describing the real limits of the used method – the IFAT, using proper references. | We modified in this way:  “IFAT and ELISA are the most commonly used serological methods. In particular, the in-house IFAT prepared with whole body parasite as antigen, following the World Organisation for Animal Health (OIE) procedure is considered the “gold standard” for serological diagnosis [1,10]. It is suitable for epidemiological studies, individual diagnosis and in treatment follow-up [10]. However, IFAT interpretation is operator-dependent and it requires trained and experienced personnel especially in case of titres around the cut-off. ELISA tests showed variable diagnostic performance depending on the type of antigen used. In particular, ELISA based on soluble promastigote and amastigote antigens had high sensitivities in both symptomatic and asymptomatic dogs, while rK39 ELISA seem to be less sensitive in detecting asymptomatic cases [11].” |
| L. 64 | Please replace the word “conclusive” by the more appropriate idea which is “strongly indicative” or something more like that. Only PCR and parasite isolation / observation are conclusive. | Thanks, we modified it. |
| L. 69-72 | Please observe that these two phrases are quite antagonistic. Both ideas can be true. Please define your line of ideas clearly, because there seems to be an implicit objective in them. Make it explicit, which can bring value to your work. Did you intent to evaluate the kennel’s conditions or possible role in the epidemiology of leishmaniasis by L. infantum? | We modified as follows: “The aim of this study was to assess the influence of different individual, environmental and spatial risk factors on the dog exposure to *L. infantum* as well as to estimate the seroprevalence among owned and kennel dogs in the Lazio region (central Italy), where canine leishmaniasis is endemic”  See also comment to L 32-34 |
| L 77 | **Materials and Methods**: In general, it requires some more information. Was there any inclusion/exclusion criteria for study dogs? | We specified some inclusion criteria for the dogs enrolled in the study:  “As inclusion criteria, only the dogs identified with a microchip were enrolled in this study. If more than one test was performed on the same animal in the study period, only the positive one or the one with the highest titre was considered. All dogs enrolled in this study had no history of previous vaccination against leishmaniasis.” |
| L. 84-85 | please rewrite the phrase. | We modified as follows:  “This is a highly heterogeneous area regarding altitude, weather conditions, land cover/use and human population density. The region is mainly hilly with a mean altitude of about 400 meters above sea level, ranging from the plain to the mountain.” |
| L. 89 | “During ‘the implementation’ of the present study”… We are in 2020. | Thanks, we modified it. |
| L. 116 and on: | Please provide information on the clinical handling of the kenneled dogs; methods/criteria for examination and sample collection. | We better specified the criteria in the ‘sampling and data’ paragraph as follows:  “Blood samples were collected for the following reasons: kennel admittance, annual screening, differential diagnosis or follow up after treatment.”  Please see also comment on L 185 |
| L. 123 | Is this questionnaire directed to whom? It is published or disclosed in some publication? Is it validated? Otherwise, perhaps it is better to refer to a detailed anamnesis. | The “questionnaire” is provided by a Law and it is compulsory filled out by a public or private veterinarian for every CanL positive case. However, we modified the text removing the word “questionnaire” and we use “data” that looks more pertinent. Thank you for the comment. |
| L. 140-141 | Please provide a little more rationale’s detail on AP and TP. | We better specified as follows: “The apparent prevalence (AP), the proportion of all those who are tested who, rightly or wrongly, test positive, and 95% confidence interval (CI) were calculated using a binomial distribution. The true prevalence (TP), estimated from AP adjusting for the diagnostic test sensitivity (Se) and specificity (Sp), was calculated using the following the Rogan and Gladen equation:  TP = (AP + Sp – 1)/(Se + Sp – 1).” |
| L. 153-156 | Please observe the policy of the Journal and the general legislation for the ethical approaches of research using animals. There was handling of animals in the kennels. They were examined and sampled. | We added sentences as follows: “The blood collection was performed in accordance with the European Legislation on Animal Welfare. Approval of an ethics committee was not required for sampling because a single blood collection sample is considered a routine procedure in domestic animals. The owned dogs were enrolled during routinely veterinary clinical activity.” |
|  | **Results**: In general, the entire section needs some language edition and a more objective organization, at least by describing related results in accordance with the analytic method used, in separate paragraphs. | We modified the text and organized the section in separate paragraphs:  - Risk factors;  - Seroprevalence |
| L. 185 | Clinical signs in relation to serology should be described in a more complete way. “Etc” is not very precise. The literature is plenty of studies and data on the close relation between susceptibility, predominant immune response, parasite load, frequency and intensity of disease manifestation, infectiousness to the vector, and serology in dogs. Please amend. Is there a possibility of co-morbidities in the region? | We removed all the parts relative to clinical signs because we did not have enough data to support inference.  Even if the veterinarians took the anamnesis and recorded for their needs the possible clinical signs, this information was transmitted to the laboratory in a small proportion of cases. Thus, we decided not to present the results in this revision.  We do not think that our data can support any relation between symptomatology and serological titres or intensity of clinical signs and parasites load. |
|  | **Discussion**: The section requires a better organization and separation of ideas by groups of related results in paragraphs. Please clearly separate literature citations from your own discussion and inputs based on your findings. | We organized the section in separate paragraphs and subparagraphs :  - Risk factors  - Individual risk factors  - Environmental risk factors  - Spatial risk factors  - Seroprevalence;  - Kennels management |
| L. 217-220 | Please explore this result with more clarity and depth, preferably in a separate paragraph, taking into consideration inclusion and exclusion criteria. This should be clear in the abstract, accordingly. | We revised it in the corresponding new lines. |
| L. 229-231 | Please elaborate this. | We modified the sentence as follows:  “In the present study, the risk of being seropositive was higher starting from 2 years old, in agreement with several studies reporting that the risk of seropositivity increased with the dog age [14,17,32-35]. This evidence could be attributed to repeated exposure to *Leishmania* during the years. On the contrary, other authors reported a bimodal age distribution of seroprevalence with one peak appearing in the young dogs (1–2 years) and a second more evident peak among the older ones (7–8 years). This pattern was explained by the composition of the target population: dogs more sensitive to the disease (e.g. the Boxer) will develop it at an early age, whereas in more resistant dogs, latent infection will not be activated until they are older and their immune system decays, often in comorbidity with other diseases [36]” |
| L. 234 and others | Leishmania should be italicized and written with an initial capital letter. Please observe it and amend it in the whole text. | Thanks, we modified it. |
| L. 241-247 | These statements are a bit vague. How is the general attitude of owners towards male or female dogs? Are males preferred in the region, for hunting or guard, for example? Would they be more exposed to infection somehow? | We modified the sentence as follows: “A significant difference in prevalence was found between sexes, with males showing higher seroprevalence. Other authors reported a higher seroprevalence in males [34,36]. This result could be due to the roaming behaviour of male dogs in rural areas or the outdoor attitude of male guard dogs, as reported in a study carried out in the study area [21]. Conversely, other studies did not found significant difference between sexes [11,37]” |
| L. 247 and on | Is the term “Conversely” appropriate here? The whole discussion about autochthonous breeds is very interesting and should be separate in another paragraph. | We wanted to say that purebred dogs are in general more at risk if compared to crossbred, except the autochthonous purebred dogs that are less at risk than crossbred. We have put this topic in a separate subparagraph. |
| L. 278-279 | Please provide a reference for the statement “Nowadays, the average increase in temperatures has likely shift the classic climatic hot zones toward higher altitudes and larger distance from the coast”... in the studied region? Otherwise, the statement becomes speculative. | We modified the paragraph and added references. |
| L. 288-290 | The statements: “For the same reason, infected dogs can be not properly treated when infected by L. infantum. In these cases, dogs may act as a source of infection for phlebotomine sand fly, in particular in overcrowded kennels and in endemic regions” – feel in disagreement with your own findings of a lower seroprevalence along time, for example, in comparison with a previous publication. The idea is valid, but please adopt a more rational insertion for this in the context of your own study and results. | We introduced this sentence to explain this apparent disagreement:  “Even if in a general decreasing trend framework of the leishmaniasis in Europe and Italy, kennels can play a role as hot spot of the disease, since they are often managed with scarce funding, the sanitary level is often low and dogs can be not properly treated with preventive treatments against ectoparasites” |
| L. 293 | Is the general welfare of dogs in kennels in the region, or particularly in the kennels you have studied, compromised? Did you observe in them any aspect that should be described in this work and discussed? Shouldn’t it then be in the objectives, if relevant? Animal welfare is obviously relevant, but since this is as objective study, data on this aspect should be treated with the same objectivity as the other scientific data. Please amend. | Animal welfare measurement was not an objective of the paper. We did not observe particular aspect to comment during the study. It was mentioned here only to state that improving the surveillance and control in CanL has a direct impact only in animal welfare at individual level. |
| L. 302 | The conclusion should be in line with the objectives and results. | Thanks, we modified it. |
| Figures and tables | All titles of figures and table should be more complete, with explanatory info enough to be understood without the reader having to go back to the text. | Thanks, we modified all the figures and the table legend |

Reviewer #2

|  | This is a very interesting paper focusing on the seroprevalence of CanL in the Mediterranean Region in Italy. Although the paper has local significance their findings are interesting especially if compared with other countries/regions. The paper is well-written and the analysis supported their findings and generated data. English is of high quality. I therefore recommend acceptance. |  |
| --- | --- | --- |

Reviewer #3

|  | The manuscript contains a well conduced analysis of seroprevalence and risk factors to canine Leishmaniasis in Lazio region. The work is well designed, contains relevant information, and it is within the scope of the journal. I have some suggestions as described below: |  |
| --- | --- | --- |
| L 104, 123 e 126 | M&M- L. 104- Organization Age classes are repeated in L. 123 and 126 . | Thanks, we modified it. |
| Figure 3 | **Results** According with text, L. 171-172 - “A bimodal relation was found between seropositivity and age: age classes 2-4 and >6 years showed higher prevalence (Fig 3).” However, in figure 3, the Y axis shows the number of seropositive dogs, this gives the false impression that there was a fall in the 5-6 age group. So, I suggest using % seropositive (seroprevalence). | As suggested, we revised the Figure 3, removing the absolute number of cases and inserting a ratio (seroprevalence) and its relative confidence intervals. |
